# Supplementary material for: Development of nano-emulsions based on Ayapana triplinervis essential oil for the control of Aedes aegypti larvae
Source: PLoS One. 2021 Jul 9;16(7):e0254225. doi: 10.1371/journal.pone.0254225 (PMC8270136; doi:10.1371/journal.pone.0254225)

**Supporting information**

**S1 Fig. Chromatogram of the essential oil of *A. triplinervis* morphotype A.**


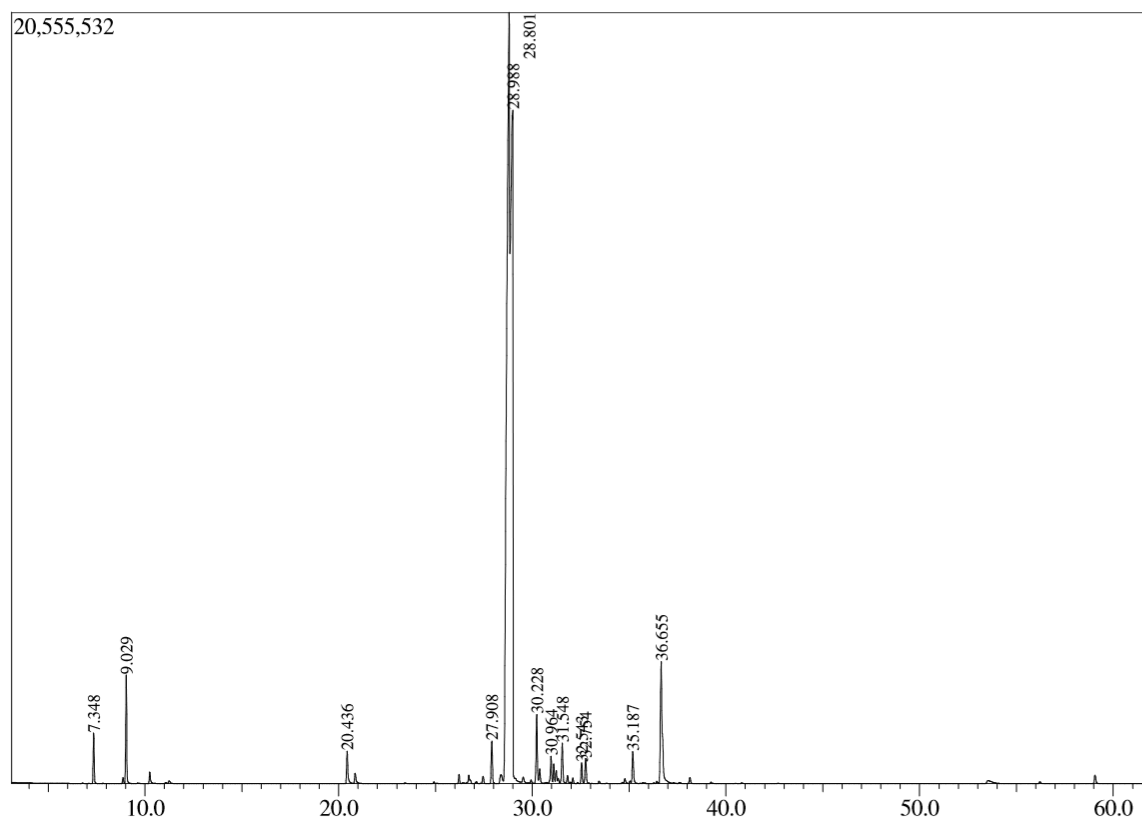

Supplement: S1 Fig — (DOCX) [file pone.0254225.s001.docx]
