# Supplementary material for: Development of nano-emulsions based on Ayapana triplinervis essential oil for the control of Aedes aegypti larvae
Source: PLoS One. 2021 Jul 9;16(7):e0254225. doi: 10.1371/journal.pone.0254225 (PMC8270136; doi:10.1371/journal.pone.0254225)
Supplement: S3 Fig — (DOCX) [file pone.0254225.s003.docx]

**Supporting information**

**S3 Fig. Physical parameters of *A. triplinervis* morphotype A nano-emulsions in different hydrophilic-lipophilic balance values at 0, 7, 14 and 21 days.**

Particle Size, polydispersivity index, zeta potencial and Electrical conductivity of nano-emulsions at different hydrophilic-lipophilic balance on day 0.

Particle Size, polydispersivity index, zeta potencial and Electrical conductivity of nano-emulsions at different hydrophilic-lipophilic balance on day 7.

Particle Size, polydispersivity index, zeta potencial and Electrical conductivity of nano-emulsions at different hydrophilic-lipophilic balance on day 14.

Particle Size, polydispersivity index, zeta potencial and Electrical conductivity of nano-emulsions at different hydrophilic-lipophilic balance on day 21.
