# Supplementary material for: Development of nano-emulsions based on Ayapana triplinervis essential oil for the control of Aedes aegypti larvae
Source: PLoS One. 2021 Jul 9;16(7):e0254225. doi: 10.1371/journal.pone.0254225 (PMC8270136; doi:10.1371/journal.pone.0254225)
Supplement: S1 Table — (DOCX) [file pone.0254225.s005.docx]

**Supporting information**

**S1 Table. Chemical composition, retention time (R_T_), percentage and calculated Linear Retention Index (LRI) that is reported in the literature [16] of essential oil of *A. triplinervis* morphotype A.**

|  | | **Morphotype A** | | |
| --- | --- | --- | --- | --- |
| **Compound** | **T_R_** | **Percent (%)** | **LRI** | **LRI Lit. [18]** |
| α-Pinene | 7.348 | 0.93 | 948 | 932 |
| β-Pinene | 9.029 | 2.16 | 943 | 974 |
| Thymol Methyl Ether | 20.436 | 0.68 | 1231 | 1232 |
| α-Gurjunene | 27.908 | 0.98 | 1419 | 1409 |
| (E)-Caryophyllene | 28.801 | 45.93 | 1494 | 1419 |
| Thymohydroquinone Dimethyl Ether | 28.988 | 32.93 |  | 1426 |
| α-Humulene | 30.228 | 1.60 | 1579 | 1552 |
| Aciphyllene | 30.964 | 0.53 | 1490 | 1501 |
| β-Selinene | 31.548 | 0.88 | 1469 | 1490 |
| α-Muurulene | 32.543 | 0.45 | 1435 | 1392 |
| γ-Gurjenene | 32.754 | 0.64 | 1461 | 1475 |
| Caryophyllene Oxide | 35.187 | 0.76 | 1506 | 1583 |
| 2,5-Di-Tert-Buthyl-1,4-Benzenoquinone | 36.655 | 4.22 |  | 1633 |
|  | | | | |
