# Supplementary material for: Development of nano-emulsions based on Ayapana triplinervis essential oil for the control of Aedes aegypti larvae
Source: PLoS One. 2021 Jul 9;16(7):e0254225. doi: 10.1371/journal.pone.0254225 (PMC8270136; doi:10.1371/journal.pone.0254225)
Supplement: S2 Table — (DOCX) [file pone.0254225.s006.docx]

**Supporting information**

**S2 Table. Chemical composition, retention time (R_T_), percentage and calculated Linear Retention Index (LRI) that is reported in the literature [16] of essential oil of *A. triplinervis* morphotype B.**

|  | | **Morphotype B** | | |
| --- | --- | --- | --- | --- |
| **Compound** | **T_R_** | **Percent (%)** | **LRI** | **LRI Lit. [18]** |
| α-Pinene | 7.352 | 1.25 | 948 | 932 |
| β-Pinene | 9.028 | 2.26 | 943 | 974 |
| α-Gurjunene | 27.890 | 0.41 | 1419 | 1409 |
| Thymohydroquinone Dimethyl Ether | 28.656 | 84.53 |  | 1424 |
| α-Humulene | 30.134 | 1.11 | 1579 | 1452 |
| β-Selinene | 31.505 | 0.41 | 1469 | 1489 |
| Valencene | 32.733 | 0.53 | 1474 | 1496 |
| Caryophyllene Oxide | 35.182 | 0.67 | 1507 | 1582 |
| 2,5-Di-Tert-Buthyl-1,4-Benzenoquinone | 36.619 | 1.82 | 1633 |  |
| α-Cedrene Epoxide | 36.700 | 0.93 | 1293 | 1574 |
