# Supplementary material for: Development of nano-emulsions based on Ayapana triplinervis essential oil for the control of Aedes aegypti larvae
Source: PLoS One. 2021 Jul 9;16(7):e0254225. doi: 10.1371/journal.pone.0254225 (PMC8270136; doi:10.1371/journal.pone.0254225)
Supplement: Attachment — Submitted filename: Review report PONE-D-20-35553.docx [file pone.0254225.s007.docx]

**Review report**

Manuscript Number: PONE-D-20-35553

Article Type: Research Article

Full Title: Development of nano-emulsions based on *Ayapana triplinervis* essential oil for the control of *Aedes aegypti* larvae

**General Comments**

Nanopesticides are formulated on the basis of different nanocarrier and the principal chemical targeted for management of insect pests. Application of the insecticides in nature bears inherent problem of affecting the non-target organisms and alteration of the ambient environment. Despite higher efficacy of the insecticides, the cost-effectiveness and the ill-effects to the environment are the primary factors impeding the wide use of the pesticides. Continuous effort in improvement of the pesticides is required to combat the mosquito menace and thus the incidence of mosquito borne diseases. Mosquitoes can exploit wide range of water bodies for breeding, and the strategies for the biological regulation vary accordingly. In majority instances, the preference of the small containers and small spaces as breeding habitats by *Aedes aegypti* calls for application of the botanicals for effective regulation. No doubt, *Ae. aegypti* can exploit various other breeding habitats, the application of the botanicals are useful depending its cost-effectiveness, including least effect on non-target organism and reasonable price. Thus the present article is useful from the viewpoint of *Ae. aegypti* regulation, where the efficacy of a nano-emulsion linked with the essential oil of *Ayapana triplinervis* was assessed. In general the methodology and results are acceptable, though, improvement in the language quality is required for the article to qualify for publication. Going through the manuscript, I feel that the article is useful and qualifies for publication. However, a revision is required in language quality and the tables and figures to promote the biological control of the dengue vectors with the nano-emulsions. The following points should be considered by the authors during the revision of the manuscript.

**Introduction section**

1] Please improve the overall language quality.

2] Consider reducing the number of paragraphs and increase clarity in the working hypothesis

3] Provide few statements on the utility of the study and prospective application. I hope you would agree that several botanicals and synthetic pesticides have been highlighted for their efficacy and use in mosquito regulation, particularly, *Ae. aegypti*  and *Ae. albopictus.* So, please emphasize the significance of the present work in the context of cost and efficacy for *Ae. aegypti* regulation.

4] Please consider additional references provided for inclusion in the introduction section.

**Methodology section**

Please provide an experimental outline in a table or through a flow diagram.

Please elaborate further the methodology mentioned in the section “Evaluation of Larvicidal Activity against *A. aegypti*’. Please highlight the number of replicates (line 180…is understandable but expand further) used for each dose.

Please expand and elaborate the statements from lines 182 through 185.

Provide a logic for using the Swiss albino mice and its significance of the study in the methods section.

Please provide reasons for not using probit analysis for deducing the median dose for the nano pesticides.

In addition, please provide a clarification about how the *Ae. aegypti* instar III larvae were identified. Mention the origin of the P generation of *Ae. aegypti.* I think it would be prudent to provide the length and weight measures of the larvae…. Please consider this suggestion. You are suggesting about the most recent method of mosquito control, so please be specific about the larvae to make the whole compilation more logical and attractive for the readers and scientist.

Why mouse was considered as non-target organism and not freshwater organisms? It would have been prudent if the freshwater organisms were considered.

**Results**

Considering the results shown in Table 3 and Table 4, it appears that these two tables indicate positive control showed 100% mortality against both morphotype A and B. The biopesticide *Bacillus thuringiensis* subspecies israelensis (BTI) was used as positive control. If positive control showed 100% mortality it can be said that it has better efficacy than that of the experimental combinations. Then what is the logic to build a larvicide which actually have lower efficacy than that of established larvicide also *Bacillus thuringiensis* don’t have any toxic effect on non targeted animal. So please provide explanation or consider alternative presentation.

Please consider application of probit analysis for the dose mortality data.

**Discussion Section**

Please consider the following articles and the relevant points as applicable for the article.

Provide a comparative account of the various botanicals and their efficacy against *Ae. aegypti* and elaborate how the application of *Ayapana triplinervis*would be useful.

Please depict the prospective utility of the present study highlighting whether the nano-emulsion bears prospect in terms of cost and the effects.

Balaure PC, Dragoş B, Gudovan D, Gudovan I. 2017. 4 - Nanopesticides: a new paradigm in crop protection. New Pesticides and Soil Sensors, 129-192. <https://doi.org/10.1016/B978-0-12-804299-1.00005-9>

Chowdhury N, Ghosh A, Chandra G. 2008.Mosquito larvicidal activities of *Solanum villosum* berry extract against the dengue vector *Stegomyia aegypti*. BMC Complementary Alternative Medicine, 8, 10 (2008).

Djiwanti SR, Kaushik S. 2019. Nanopesticide: Future Application of Nanomaterials in Plant Protection. In: Prasad R. (eds) Plant Nanobionics. Nanotechnology in the Life Sciences. 255-298 pp.Springer, Cham. <https://doi.org/10.1007/978-3-030-16379-2_10>

Facknath S, Lalljee B. 2008. Study of various extracts of *Ayapana triplinervis* for their potential in controlling three insect pests of horticultural crops. Tropicultura, 26(2): 119 – 124.

Ghosh A, Chowdhury N, Chandra G. 2012. Plant extracts as potential mosquito larvicides. Indian Journal of Medical Research, 135:581-598.

Kah M, Kookana RS, Gogos A, Bucheli TD. 2018. A critical evaluation of nanopesticides and nanofertilizers against their conventional analogues. Nature Nanotechnology, 13: 677–684. https://doi.org/10.1038/s41565-018-0131-1

Kookana RS, Boxall ABA, Reeves PT, Ashauer R, Beulke S, Chaudhry Q, Cornelis G, Fernandes TF, Gan J, Kah M, Lynch I, Ranville J, Sinclair C, Spurgeon D, Tiede K, Van den Brink PJ. 2014. Nanopesticides: Guiding Principles for Regulatory Evaluation of Environmental Risks. Journal of Agricultural and Food Chemistry, 62 (19): 4227-4240. DOI: 10.1021/jf500232f

Priyanka P, Kumar D, Yadav K, Yadav A. 2019. Nanopesticides: Synthesis, Formulation and Application in Agriculture. In: Abd-Elsalam K., Prasad R. (eds) Nanobiotechnology Applications in Plant Protection. Nanotechnology in the Life Sciences. 129-143 pp. Springer, Cham. https://doi.org/10.1007/978-3-030-13296-5_7

Rawani A, Chowdhury N, Ghosh A, Laskar S, Chandra G. 2013. Mosquito larvicidal activity of *Solanum nigrum* berry extracts. Indian J Med Research,137:972-976.

Rawani A, Ghosh A, Chandra G. 2014.Laboratory evaluation of molluscicidal & mosquito larvicidal activities of leaves of *Solanum nigrum* L. Indian Journal of Medical Research, 140:285-295.

Stadler T, Butelar M, Valdez SR, Gitto JG. 2017. Particulate nanoinsecticides: a new concept in insect pest management. DOI: 10.5772/intechopen.72448

Hope these suggestions are useful in enhancement of the manuscript.

Thank you.
