# Supplementary material for: Development of nano-emulsions based on Ayapana triplinervis essential oil for the control of Aedes aegypti larvae
Source: PLoS One. 2021 Jul 9;16(7):e0254225. doi: 10.1371/journal.pone.0254225 (PMC8270136; doi:10.1371/journal.pone.0254225)
Supplement: Attachment — Submitted filename: Response to Reviewers (1).docx [file pone.0254225.s008.docx]

Ahmed Ibrahim Hasaballah

Academic Editor

PLOS ONE

Dear Academic Editor,

We are writing to respond to reviewers for the requested adjustments to the manuscript entitled “Development of nano-emulsions based on *Ayapana triplinervis* for the control of *Aedes aegypti* larvae”.

JOURNAL REQUIREMENTS:

**1) Please ensure that your manuscript meets PLOS ONE's style requirements, including those for file naming.**

Response: The style was revised according to the guidance.

**2) In your Methods section, please provide additional information regarding the permits you obtained for the work.**

Responde: The number of the document authorizing access to the genetic heritage has been added to the methodology.

REVIEWER #1:

- **Mice were used in the study, thus an Ethics Statement is required by PLOS.**

Response: The approval code of the ethics and animal research committee was provided on lines 197 and 198. Details of Humane endpoints were provided in a specific form of the Journal in the submission.

- **16-17: Two symbols are used to indicate equal contribution and each author has one or the other. This suggests that all authors contributed equally and only one symbol is needed.**

Response: adjusted according to the reviewer's request.

- **26: not necessary to capitalize Gas Chromatography or Mass Spectrometry**

Response: adjusted according to the reviewer's request.

- **27-30: I don’t think it necessary to cite the protocols used for larval bioassay and toxicity in the abstract.**

Response: adjusted according to the reviewer's request.

- **31-33: Define what % is referring to (assuming it’s of the total but is it by mass, volume, etc).**

Response: adjusted according to the reviewer's request.

- **34: PdI and ZP are non-standard acronyms and may need to be defined for readers to know their meaning.**

Response: adjusted according to the reviewer's request.

- **47-67: Extensive introduction of DENV, CHIKV and ZIKV is provided but are not highly relevant to the study. Recommend reducing the extent of the introduction of these topics to simply state they are transmitted by Ae. aegypti.**

Response: adjusted according to the reviewer's request.

- **77: sentences that start with a species name should not use abbreviation**

Response: adjusted according to the reviewer's request.

- **Methods: all equipment and reagents used in the study should include the manufacturer and location of the company**

Response: adjusted according to the reviewer's request.

- **197: PLOS may require IACUC protocol or approval number to be included in the manuscript.**

Response: (CEP – Unifap – 004/2019), in line 190, is the approval number by IACUC.

- **207: What does “03” refer to?**

Response: Experimental grups: MAEON; MBEON; and Negative Control.

- **229: Please describe how the tissues were routinely processed.**

Response: adjusted according to the reviewer's request.

- **230: State microscope brand, model, etc.**

Response: adjusted according to the reviewer's request between lines 224 and 226

- **239: MAEO is not previously defined**

Response: adjusted according to the reviewer's request.

- **240: Should E be replaced with “Beta”?**

Response: adjusted according to the reviewer's request.

- **256-291: This is discussion that should be moved to Discussion. Note that the Discussion is already overly lengthy.**

Response: adjusted according to the reviewer's request.

- **309: Please state in the Methods how the emulsions were stored for the 21 day study.**

Responde: Lines 159 to 160.

- **Table 2: Only particle size is mentioned in the manuscript outside of this table. The other physical characteristics should be described elsewhere or removed if not of great relevance to the study.**

Response: We confirm the importance of physical and chemical characterization. Otherwise, we emphasize the contextualization of particle size, polydispersivity index and zeta potential between lines 330 to 350; electrical conductivity between lines 355 to 361. As for electrophoretic mobility, we chose to exclude such data as suggested by the reviewer.

- **Fig 2 and 3: Fonts for x- and y-axis are too small to view.**

Response: adjusted according to the reviewer's request in lines 321-322; and 332-333

- **333-347: Please integrate into the Discussion**
- Response: adjusted according to the reviewer's request.
- **345: O/W (and later, W/O) is not defined**

Response: adjusted according to the reviewer's request.

- **359-360: Please provide data that supports this conclusion.**

Response: adjusted according to the reviewer's request.

- **Table 3 and 4: This data would be much easier to interpret were it presented as a scatter plot with linear regression line fitted to the data. Alternatively, these tables could be removed as the LC50 and LC90 data provided in Table 5 may be sufficient for demonstrating the effect of the extracts on larval mortality. If these tables are retained (not recommended), please state what the dots and asterisk on 24 h and 48 h indicate.**

Response: Dots and asterisks indicate statistical differences on the same line. The meaning is in lines 367 and 368 below the column.

- Table 5: Statistical analysis of MAEO vs MAEON and 24h vs 48 h should be conducted.

Response: adjusted according to the reviewer's request.

- **386: Incorrect statement: LC90 for MBEO and MBEON at 48h was higher than 24h. These differences should be described in the manuscript.**

Response: adjusted according to the reviewer's request.

- **394-396: There is not sufficient data to draw this conclusion (the most prevalent component may not have the highest biological activity).**

Response: adjusted according to the reviewer's request.

- **397-399: Please indicate how this study contradicts what is presented in the manuscript.**

Response: The opposite condition is related to the high content of Thimohydroquinone Dimethyl Ether in morphotype B that showed high larvicidal activity, different from the studies indicated in the reference that presented moderate larvicidal and adulticidal activity of Eupatorium capillifolium that presents with Thymohydroquinone Dimethyl Ether as one of the major compound.

- **404-406: This statement is not well supported by the data.**

Response: The Declaration has been corrected.

- **408 - 410: Please provide data that supports the rationale for selecting 100 ug/ml as a value for drawing comparisons (it seems arbitrary).**

Response: The LC50 comparison is not arbitrary and is in accordance with the classification proposed by Cheng et al (2003), as mentioned in the paragraph.

- **413-415: Field trials of the emulsions would need to be conducted to draw this conclusion.**

Response: The Declaration has been corrected.

- **418: The surface areas of the structures for the controls and treatments should be measured to draw meaningful conclusions that are supported by the data. Samples were fixed in 70% ethanol which would likely deform the structures and reduce their volume. Notably, Figure 4D is indicated as a control, but appears to be more deformed than the treatment displayed in Figure 4E.**

Response: The item was adjusted according to what was requested. SEM analyzes were removed to avoid compromising the paper. The result was described according to the new context

- **419: Please provide arrowheads in Fig4 that show where epithelial tissue is peeling.**

Response: adjusted according to the reviewer's request.

- **Fig 4: Scale bars must be included for each image.**

Response: adjusted according to the reviewer's request.

- **459: Text states there is a significant decrease in liver weight, the weight of the liver of male mice treated with MBEON was higher than control (an increase). Please explain.**

Response: adjusted according to the reviewer's request. The explanation has been added to the text on lines 425-432 .

- **Discussion: as noted previously, the Discussion is overly lengthy and relatively unfocused. Many paragraphs are a single run-on sentence. Please revise to improve focus.**

Response: adjusted according to the reviewer's request. Some paragraphs have been deleted

- **523: Please provide evidence that the larvicide is eco-friendly.**

Response: A reference was added to the item to support the conclusion.

- **622 and elsewhere: The convention is to use commas for separating thousands and periods to signify the decimal place in a value.**

Response: adjusted according to the reviewer's request.

- **Supplementary Tables 7 – 12: These tables are not highly relevant as the results are summarized in Table 5. However, if they are retained, the number of significant digits must be reduced to reflect the actual sample size (mortality was unlikely measured to the thousandths level of precision).**

Response: The tables were removed according to the reviewer's request.

- **S15: These images should be analyzed (e.g. structures measured), annotated (e.g. arrowheads) and have scale bars or removed from the manuscript.**

Response: The figures were removed according to the reviewer's request.

REVIEWER #2:

**Introduction section**

**1] Please improve the overall language quality**.

Response: The tables were removed according to the reviewer's request.

**2] Consider reducing the number of paragraphs and increase clarity in the working hypothesis 3] Provide few statements on the utility of the study and prospective application. I hope you would agree that several botanicals and synthetic pesticides have been highlighted for their efficacy and use in mosquito regulation, particularly, Ae. aegypti and Ae. albopictus. So, please emphasize the significance of the present work in the context of cost and efficacy for Ae. aegypti regulation.**

**Response:**

Response: adjusted according to the reviewer's request.

**4] Please consider additional references provided for inclusion in the introduction section**

- The following references have been added to the manuscript:Kookana RS, Boxall AB, Reeves PT, Ashauer R, Beulke S, Chaudhry Q, et al. Nanopesticides: guiding principles for regulatory evaluation of environmental risks. J Agric Food Chem. 2014; 62: 4227-4240. doi: [10.1021/jf500232f](https://doi.org/10.1021/jf500232f)
- Kah M, Kookana RS, Gogos A, Bucheli, TD. A critical evaluation of nanopesticides and nanofertilizers against their conventional analogues. Nat Nanotechnol*.* 2018, 13: 677-684. doi: 10.1038/s41565-018-0131-1
- Silvério MRS, Espindola LS, Lopes NP, Vieira PC. Plant natural products for the control of *Aedes aegypti*: The main vector of important arboviruses. Molecule. 2020, 25: 3484 - 3526. doi: 10.3390/molecules25153484
- Badawi MS. Histological study of the protective role of ginger on piroxicam-induced liver toxicity in mice. J Chin Med Assoc*.* 2019, 82:11-18. doi: 10.1016/j.jcma.2018.06.006
- Yuan L, Kaplowitz N. Mechanisms of drug-induced liver injury. Clin Liver Dis. 2013, 17: 507-518. doi: 10.1016/j.cld.2013.07.002

**Please elaborate further the methodology mentioned in the section “Evaluation of Larvicidal Activity against A. aegypti’. Please highlight the number of replicates (line 180…is understandable but expand further) used for each dose**

**Please provide reasons for not using probit analysis for deducing the median dose for the nano pesticides.**

Response: Probit analysis was used to deduct the LC50 for essential oil and nanoemulsion of each morphotype of the plant species, as shown in Table 5.

**In addition, please provide a clarification about how the Ae. aegypti instar III larvae were identified. Mention the origin of the P generation of Ae. aegypti. I think it would be prudent to provide the length and weight measures of the larvae…. Please consider this suggestion. You are suggesting about the most recent method of mosquito control, so please be specific about the larvae to make the whole compilation more logical and attractive for the readers and scientist.**

Response: The information was added on lines 157-158

**Why mouse was considered as non-target organism and not freshwater organisms? It would have been prudent if the freshwater organisms were considered.**

Response: Mice were considered in this study as non-target organisms due to the anthropophilic characteristics of Ae aegypti. When developing in urban breeders, the direct application of a nanoinsecticide can be ingested through the consumption of water by different domestic animals and even humans. However, future studies may be conducted to assess toxicity in freshwater organisms.

Results

**Considering the results shown in Table 3 and Table 4, it appears that these two tables indicate positive control showed 100% mortality against both morphotype A and B. The biopesticide Bacillus thuringiensis subspecies israelensis (BTI) was used as positive control. If positive control showed 100% mortality it can be said that it has better efficacy than that of the experimental combinations. Then what is the logic to build a larvicide which actually have lower efficacy than that of established larvicide also Bacillus thuringiensis don’t have any toxic effect on non targeted animal. So please provide explanation or consider alternative presentation. Please consider application of probit analysis for the dose mortality data.**

Response: BTI, as a positive control, was used as a parameter to compare the magnitude of larvicidal activity of A. triplinervis nanoformulations. Despite showing a higher percentage, BTI alone is not able to effectively supply all public policies for the control of Aedes aegypti. In this sense, the nanoformulations of A. triplinervis can be inserted as an auxiliary integrative practice in the control of Ae aegypti. Allied to this point, this study has additional value when it demonstrates the phytochemical profile of essential oils of the species' morphotypes exclusively in the scientific literature. Therefore, we consider A. triplinervis nanoformulations with scientific and biological value within the proposal presented.

Discussion Section

**Please consider the following articles and the relevant points as applicable for the article. Provide a comparative account of the various botanicals and their efficacy against Ae. aegypti and elaborate how the application of Ayapana triplinerviswould be useful. Please depict the prospective utility of the present study highlighting whether the nano-emulsion bears prospect in terms of cost and the effects.**

Response: We reorganized the paragraphs to meet the requested demand and inserted some of the references to clarify the ideas.

Thank you for your time and consideration to improve this manuscript.

Sincerely,

The autors
