# Supplementary material for: Development of nano-emulsions based on Ayapana triplinervis essential oil for the control of Aedes aegypti larvae
Source: PLoS One. 2021 Jul 9;16(7):e0254225. doi: 10.1371/journal.pone.0254225 (PMC8270136; doi:10.1371/journal.pone.0254225)
Supplement: Attachment — Submitted filename: Response to Reviewers (2nd round).docx [file pone.0254225.s009.docx]

Ahmed Ibrahim Hasaballah

Academic Editor

PLOS ONE

Dear Academic Editor,

We are writing to respond to reviewers for the requested adjustments to the manuscript entitled “PONE-D-20-35553R1 Development of nano-emulsions based on *Ayapana triplinervis* for the control of *Aedes aegypti* larvae”

JOURNAL REQUIREMENTS:

1) **Please review your reference list to ensure that it is complete and correct. If you have cited papers that have been retracted, please include the rationale for doing so in the manuscript text, or remove these references and replace them with relevant current references. Any changes to the reference list should be mentioned in the rebuttal letter that accompanies your revised manuscript. If you need to cite a retracted article, indicate the article’s retracted status in the References list and also include a citation and full reference for the retraction notice.**

RESPONSE: The references were analyzed in detail according to the requirements of the scientific journal. The changes were highlighted in the manuscript, when necessary.

REVIEWER #1:

- **The authors may have not noted the concern by this reviewer with the mass spectra chromatograms that are included in the Supplementary Data as there was no author response to this point.
  The concern noted in the review is here: "Notably, the Supplementary Data contains mass spectra chromatograms that appear to have been published elsewhere (e.g., by NIST and Adams (2017)). I doubt that PLOS permits the republishing of figures. I recommend the authors remove those figures and either replace them with mass spectra of standards that they generate for each chemical that was analyzed or state the diagnostic fragment ions of each chemical they analyze that match what is reported in the literature." Please respond to the above comment and make any needed changes to the manuscript that are appropriate.**

RESPONSE: We regret that this adjustment in the manuscript has gone unnoticed by our team. Receive our sincere apologies! We inform that the chromatograms were removed from the supplementary material, as instructed by the reviewer.

- **Authors did not include the scale bars in Fig. 4 that was recommended by the reviewer.**

RESPONSE: The scale bar was added to figure 4, according to the reviewer's guidance.

Additionally, we inform that the word nanoemulsion was standardized for nano-emulsion in the manuscript and in the supporting information.

Thank you for your time and consideration to improve this manuscript.

Sincerely,

The autors
